# Turkish adaptation and psychometric validation of the Normative Male Alexithymia Scale–Brief Form (NMAS-BF) among male university students

**DOI:** 10.3389/fpsyg.2026.1851206

**Published:** 2026-08-27

**Authors:** İrem Özel

**Affiliations:** 1 Department of Nursing, Faculty of Health Sciences, Acibadem Mehmet Ali Aydinlar University, Istanbul, Türkiye; 2 Department of Nursing, Faculty of Health Sciences, Eskisehir Osmangazi University, Eskisehir, Türkiye

**Keywords:** alexithymia, emotional restriction, masculine socialization, masculinity, normative male alexithymia, psychometrics, Turkish male university students

## Abstract

**Introduction:**

Normative male alexithymia reflects masculinity-related difficulties in identifying and expressing emotions and is theoretically linked to restrictive emotionality within masculine socialization. Existing measures predominantly assess clinical levels of alexithymia and may fail to capture subclinical, culturally embedded patterns of emotional restriction associated with masculine socialization. This study aimed to adapt the Normative Male Alexithymia Scale–Brief Form (NMAS-BF) to Turkish language and culture and to evaluate its psychometric properties.

**Methods:**

A methodological study was conducted with Turkish male university students. Linguistic equivalence was examined in a bilingual sample (*n* = 46), followed by a pilot study (*n* = 30). Separate non-overlapping samples were used for exploratory factor analysis (*n* = 376) and confirmatory factor analysis (*n* = 376) to assess structural validity. Exploratory factor analysis was conducted using principal axis factoring. Internal consistency was evaluated using Cronbach’s alpha, corrected item-total correlations, and alpha-if-item-deleted values.

**Results:**

The Turkish version of the NMAS-BF demonstrated high internal consistency (α = 0.890), with corrected item-total correlations ranging from 0.617 to 0.770. Exploratory factor analysis using principal axis factoring supported a single-factor structure explaining approximately 58.09% of the common variance. Confirmatory factor analysis indicated good model fit for the one-factor model, χ^2^(9) = 20.66, χ^2^/df = 2.30, RMSEA = 0.059, CFI = 0.99, TLI/NNFI = 0.99, SRMR = 0.025, and GFI = 0.98.

**Conclusion:**

The findings provide initial evidence for the internal consistency and structural validity of the Turkish NMAS-BF among Turkish male university students. Beyond its psychometric properties, the scale provides a culturally adapted tool for examining masculinity-related emotional restriction within the context of Turkish masculine socialization. Future studies may use the Turkish NMAS-BF to investigate associations between emotional restriction, emotional awareness, emotional expression, and psychological functioning among more diverse samples of Turkish men.

## Highlights

- Normative male alexithymia reflects masculinity-related emotional restriction.
- The Turkish NMAS-BF showed high internal consistency among male university students.
- Exploratory and confirmatory factor analyses supported a one-factor structure.
- The adaptation is contextualized within Turkish masculine socialization.
- The Turkish NMAS-BF may support future research on masculinity-related emotional restriction.

## 1 Introduction

In literal terms, alexithymia means “emotions without words”. In clinical use, alexithymia refers to “not being able to put emotions into words”. Alexithymia is a personality trait that involves various deficiencies in the cognitive and affective processing of emotions (Levant et al., 2014), and is increasingly conceptualized as a multidimensional construct including emotional awareness deficits, difficulties in emotional expression, and externally oriented thinking styles (Stiller et al., 2026).

Levant et al. (2006) used the term “normative male alexithymia” to describe the observed limitations of men in recognizing, identifying, and particularly expressing emotions (Levant et al., 2006). As a result of childhood socialization experiences, men are discouraged from expressing vulnerability and attachment-related emotions and from developing a vocabulary or awareness related to many of these emotions (Levant and Parent, 2019), a process that is reinforced through familial and cultural gender role expectations and masculine norm adherence (Shaked et al., 2026; Tintori et al., 2026). Recent studies indicate that conformity to masculine norms significantly predicts alexithymic traits through mechanisms such as reduced empathy, emotional restriction, and limited distress disclosure among men (Kantar et al., 2026; Likhitha and Sutar, 2026).

In the present study, normative male alexithymia is theoretically framed as a manifestation of restrictive emotionality shaped by masculine role socialization. Following Levant and Parent’s conceptualization, the present study treats normative male alexithymia as theoretically linked to restrictive emotionality, while acknowledging that masculine socialization itself was not directly measured in the present data (Levant and Parent, 2019). This framework assumes that emotional restriction is not merely an individual deficit but is also learned and reinforced through gendered expectations concerning strength, self-control, autonomy, and avoidance of vulnerability. Within this framework, the NMAS-BF is considered an operational measure of masculinity-related emotional restriction, as its items assess difficulties in recognizing, naming, and expressing emotions. The theoretical logic of the present study is therefore as follows: masculine role socialization may reinforce restrictive emotionality; restrictive emotionality may limit men’s ability to recognize, name, and express emotions; and these difficulties are operationalized by the NMAS-BF as normative male alexithymia.

The Turkish context provides a particularly relevant setting for examining normative male alexithymia because masculine identities in Türkiye are shaped by the interaction of traditional gender expectations, family-based socialization, social respectability, and ongoing social change. Research on masculinities in Türkiye indicates that being perceived as a “real man” is commonly associated with strength, self-control, responsibility, authority, and the avoidance of vulnerability. Although masculinities in Türkiye are not homogeneous, men frequently negotiate masculine identity through expectations of emotional toughness, control, and social respectability. These culturally embedded expectations may discourage emotional disclosure and reinforce the perception that vulnerability is inconsistent with masculine identity (Boratav et al., 2014; Keskin, 2018; Tekkas Kerman and Betrus, 2019). In this context, Turkish masculine socialization can be conceptualized through several interrelated dimensions, including family-based expectations of responsibility and authority, social norms emphasizing emotional toughness and self-control, ideals of honor and respectability, and peer-based reinforcement of avoiding vulnerability. These dimensions are theoretically relevant to normative male alexithymia because they may discourage the recognition, verbalization, and open expression of emotions.

This cultural background is theoretically compatible with the structure of the NMAS-BF. Conceptually, the six-item structure of the NMAS-BF corresponds to three closely related aspects of restrictive emotionality: difficulty recognizing internal emotional states, difficulty naming emotions, and difficulty expressing emotions to others. These aspects are consistent with masculine norms emphasizing emotional control, toughness, self-reliance, and avoidance of vulnerability. Previous Turkish studies have shown that masculinity, femininity, gender role conflict, and male role norms can be meaningfully examined in Turkish samples. Accordingly, adapting the NMAS-BF into Turkish provides an opportunity to assess masculinity-related restrictive emotionality within the Turkish cultural context (Boratav et al., 2014; Lease et al., 2009; Lease et al., 2013; Özkan and Lajunen, 2005).

Although normative male alexithymia has been discussed in relation to men’s emotional functioning and help-seeking processes, the present study does not examine clinical outcomes, help-seeking behavior, or mental health service utilization. Existing alexithymia measures primarily assess general or clinically elevated alexithymic traits, whereas the NMAS-BF was developed to assess subclinical, masculinity-related emotional restriction. This distinction is consistent with the development of the NMAS-BF as a brief measure of men’s gender-socialized limitations in emotional expression, informed by the masculine norm of restrictive emotionality rather than by clinical alexithymia alone (Levant and Parent, 2019; Levant et al., 2006). Although normative male alexithymia is theoretically grounded in masculine role socialization, the present study did not directly measure masculine socialization. Therefore, consistent with the caution noted in the original NMAS-BF development study, the findings should not be interpreted as direct evidence that Turkish masculine socialization causes normative male alexithymia. Accordingly, a Turkish adaptation of the NMAS-BF is needed to evaluate whether this brief measure can reliably and validly assess normative male alexithymia within the Turkish cultural context (Boratav et al., 2014; Levant and Parent, 2019; Levant et al., 2006; Tekkas Kerman and Betrus, 2019).

The present study aimed to adapt the Normative Male Alexithymia Scale–Brief Form (NMAS-BF) into Turkish and to evaluate its psychometric properties among male university students. A secondary aim was to contextualize normative male alexithymia within the literature on Turkish masculine socialization, particularly in relation to culturally embedded expectations concerning emotional restriction, emotional awareness, and emotional expression. The cultural discussion in this study is used to contextualize the adaptation of the NMAS-BF rather than to test causal, predictive, or explanatory relationships between masculine socialization and alexithymia.

## 2 Materials and methods

### 2.1 Study design

This study employed a cross-sectional methodological design.

### 2.2 Setting and procedure

Study preparation and permission procedures were conducted between May 2019 and November 2019. Data collection began only after ethics committee approval (October 7, 2019) and institutional approval (November 15, 2019) had been obtained, and was conducted between November 2019 and May 2020 with male students studying at a university in Eskişehir.

### 2.3 Participants

The study population comprises male students studying at a university in Eskişehir (*N* = 17,857). The sample size was calculated as 376 participants based on a known population. Participants were selected via proportional stratified sampling. According to this calculation, the condition that the sample should be at least 5–10 times the number of scale items to perform a factor analysis of scale reliability and validity studies is also ensured.

Inclusion criteria for volunteers

1. Being enrolled as a student at the university where the study was conducted
2. Self-identifying as male
3. Voluntarily agreeing to participate in the study
4. Being 18 years of age or older
5. Having sufficient Turkish language proficiency to complete the questionnaire
6. Providing complete responses to the NMAS-BF items

The sample was restricted to male university students for both theoretical and methodological reasons. Theoretically, the NMAS-BF was developed to assess normative male alexithymia, a masculinity-related construct; therefore, self-identification as male was necessary for evaluating the Turkish adaptation of a male-specific measure. Methodologically, university students were selected to obtain a relatively homogeneous sample in terms of educational context and developmental period during the initial psychometric validation process. Such homogeneity may reduce uncontrolled variability related to age, educational background, and life-course differences while allowing the factor structure of the adapted scale to be examined more clearly. In addition, emerging adulthood is a developmental period in which masculine norms and emotional expression remain particularly salient. However, this sampling strategy limits generalizability, and the findings should be interpreted as applying primarily to Turkish male university students rather than to all Turkish men.

No additional exclusion criteria based on psychiatric history, current mental health status, or prior psychological treatment were applied, as the study was designed as an initial psychometric adaptation study rather than a clinical comparison study.

Proportional stratified sampling was performed according to educational level, including associate degree, undergraduate, master’s, and doctoral students. The number of participants selected from each stratum was determined in proportion to its representation in the university population. This procedure was used to preserve the educational-level distribution of the study population within both the EFA and CFA samples.

### 2.4 Measures

The data were collected via a demographic questionnaire created for the participants and the NMAS-BF.

#### 2.4.1 Sociodemographic data form

The demographic questionnaire included questions assessing participants’ individual characteristics, including age, place of birth, number of siblings, family type, education level, economic status, and self-reported sexual interest. The sexual interest item was reported only to describe the sample and to compare the EFA and CFA samples demographically; it was not used in scoring the NMAS-BF or in the reliability, EFA, or CFA analyses. These questions were created by the researcher as a result of a review of the literature.

#### 2.4.2 Normative Male Alexithymia Scale–Brief Form (NMAS-BF)

As a result of the exploratory factor analysis (EFA) and confirmatory factor analysis (CFA) conducted by Levant et al. (2006) to evaluate the limitations of the emotional expression of males, the normative male alexithymia scale consists of a single factor containing 20 items with a factor load above 0.40 (Levant et al., 2006). The normative male alexithymia scale scores of men displayed very good internal consistency (0.92) and test-retest reliability (0.91) for 1–2 months. Levant and Parent (2019) have repeated validity and reliability analyses, as more than 10 years have passed since the development of the Normative Male Alexithymia Scale (Levant and Parent, 2019). In a study conducted with 505 men aged between 19 and 73, a 6-item model of the 7-point Likert-type (1 = Strongly disagree, 7 = Strongly agree) NMAS-BF was developed. The Cronbach’s alpha value of the items was found to be 0.80, and the use of the NMAS-BF was supported. Higher mean scores indicate higher levels of normative male alexithymia. The two reverse-worded items of the NMAS-BF, corresponding to original NMAS items N2R and N7R, were reverse-coded before calculating the total score. In the Turkish item order used in this study, these corresponded to Items 3 and 5.

### 2.5 Data collection process

#### 2.5.1 Adaptation process

A linguistic equivalence study was conducted to determine whether there were any errors in the Turkish expression of each test item and to what extent these items reflected their meanings. The following steps were followed to determine the level of linguistic equivalence.

#### 2.5.2 Translation – backtranslation and evaluation process

The original scale was translated into Turkish by three academicians whose native language is Turkish, who speak English at the C2 level, who are PhD graduates from various universities, and who are studying at schools of foreign languages. The Turkish forms that were translated were then merged into a single Turkish form by the researcher. This Turkish form was then translated back into English by translators who speak Turkish at the C2 level and whose native tongue is English. The back-translated form and the original form were sent to the researchers who developed the scale to discuss whether there were any shifts in meaning. The opinions of the field experts were obtained to determine whether the expressions of the scale were understandable and covered the subject in question. The final form of the scale was prepared.

#### 2.5.3 Evaluation of the translation

The Turkish form was reviewed together with a 4-person group of field specialists. On the basis of the criteria to express Turkish in the best possible manner, the items that seemed to be problematic were reconsidered. The final Turkish form was reached by making the necessary arrangements in line with the opinions of the experts.

#### 2.5.4 Linguistic equivalence and reliability process

The Turkish and English forms, which were obtained, were applied twice to a group of 46 male students studying in the Department of English Language Teaching at the Faculty of Education of a university in Eskişehir, with an interval of 20 days. The Kolmogorov–Smirnov/Shapiro–Wilk values were examined for the linguistic equivalence normality study. As a result of these examinations, the sample data did not display a normal distribution for the linguistic equivalence study. Further investigations were continued nonparametrically. The Wilcoxon signed-rank test was performed to compare the mean scores of the items in the Turkish and English forms. Spearman correlation tests were performed to compare item-level and total scores between the Turkish and English forms. The Spearman correlation coefficients are expected to be significant, whereas the Wilcoxon test results are not. The linguistic equivalence results are presented in Table 1.

**TABLE 1:** Linguistic equivalence results for the Turkish and English forms of the NMAS-BF (*n* = 46).

Item pair	Spearman’s r	*p*	Wilcoxon Z	*p*
Item 1 Turkish & Item 1 English	0.844	<0.001	−0.641	0.521
Item 2 Turkish & Item 2 English	0.760	<0.001	−0.070	0.944
Item 3 Turkish & Item 3 English	0.838	<0.001	−1.238	0.216
Item 4 Turkish & Item 4 English	0.855	<0.001	−0.467	0.641
Item 5 Turkish & Item 5 English	0.856	<0.001	−0.907	0.364
Item 6 Turkish & Item 6 English	0.837	<0.001	−1.665	0.096

r = Spearman correlation coefficient. Z = Wilcoxon signed-rank test. Significant correlations and non-significant Wilcoxon tests indicate linguistic equivalence.

At the stage of adaptation to the Turkish language and culture, 30 participants, who spoke both languages and who were selected using the sampling method for the NMAS-BF, were used for both the pilot study and the interpretation of the translation. In this case, both the original and Turkish versions of the scale were presented to the participants. The participants were asked to read the question aloud and provide a brief explanation concerning the meaning of each item. If an item was not easy to understand, the respondent was asked how the problem could be expressed in a different way. In this way, it was guaranteed that the matter would be understood equally by each individual. Thus, any points overlooked by translators and the panel of experts were revealed during the pilot study.

#### 2.5.5 EFA and CFA process

After the final form obtained was placed online via the Google Forms application, it was sent to 17,857 male students in the form of individual messages via the university’s course management system. At the same time, academicians, who provided associate/undergraduate/graduate education at the relevant university, were asked to share the link of the study with the students. A total of 1,760 students completed the online questionnaire; however, the analyses were restricted to the predetermined proportional stratified EFA and CFA samples to maintain consistency with the planned sampling design. The EFA and CFA subsamples were selected from the respondent pool according to the proportional stratified sampling plan based on academic unit and educational level. In this plan, strata were defined by undergraduate faculties, associate-degree vocational schools, graduate institutes, and specialty programs, and participants were allocated to each stratum in proportion to the number of eligible male students in that stratum. Separate subsamples were used to avoid evaluating the exploratory and confirmatory factor structures on the same data. The EFA and CFA analyses were conducted using two independent non-overlapping datasets, each including 376 participants. The EFA dataset was used only for exploratory factor analysis, whereas the CFA dataset was used only to test the one-factor model. Responses outside the predetermined proportional stratified samples were not included in the psychometric analyses. Considering the brief six-item structure of the NMAS-BF, the EFA sample size exceeded commonly recommended participant-to-item ratios for factor analysis. The demographic characteristics of the EFA and CFA samples are presented in Table 2. Participants within each stratum were selected randomly from the respondent pool.

**TABLE 2:** Demographic characteristics and comparison of the independent EFA and CFA samples.

Variable	EFA n (%)	CFA n (%)	χ^2^(df)	p
What is your year of birth?			χ^2^(3) = 3.19	0.363
2000 and above	111 (29.5)	118 (31.4)		
1995–1999	218 (58.0)	205 (54.5)		
1990–1994	27 (7.2)	38 (10.1)		
1989 and below	20 (5.3)	15 (4.0)		
What is your place of birth? (Regional)			χ^2^(7) = 18.10	0.012
Mediterranean Region	44 (11.7)	35 (9.3)		
Eastern Anatolia Region	22 (5.9)	12 (3.2)		
Aegean Region	45 (12.0)	54 (14.4)		
Southeastern Anatolia Region	23 (6.1)	12 (3.2)		
Central Anatolia Region	134 (35.6)	118 (31.4)		
Marmara Region	66 (17.6)	97 (25.8)		
Black Sea Region	29 (7.7)	40 (10.6)		
Outside the Borders of Türkiye	13 (3.5)	8 (2.1)		
What is the total number of siblings, including you?			χ^2^(2) = 2.59	0.274
1	24 (6.4)	31 (8.2)		
2	182 (48.4)	195 (51.9)		
3 and above	170 (45.2)	150 (39.9)		
How would you describe your family type?			χ^2^(2) = 0.77	0.679
Nuclear family	294 (78.2)	287 (76.3)		
Extended family	50 (13.3)	50 (13.3)		
Fragmented family	32 (8.5)	39 (10.4)		
What education level do you currently study at?			χ^2^(2) = 0.00	1.000
Associate Degree	32 (8.5)	32 (8.5)		
Undergraduate	297 (79.0)	297 (79.0)		
Postgraduate	47 (12.5)	47 (12.5)		
How would you describe your economic situation?			χ^2^(2) = 6.85	0.033
Income less than expense	65 (17.3)	88 (23.4)		
Income equal to expense	237 (63.0)	203 (54.0)		
Income is more than expense	74 (19.7)	85 (22.6)		
How would you describe your sexual interest?			χ^2^(4) = 6.81	0.146
I’m interested in women	346 (92.0)	349 (92.8)		
I’m interested in men	19 (5.1)	10 (2.7)		
I’m interested in both men and women	7 (1.9)	8 (2.1)		
Other/combined category as originally worded	2 (0.5)	1 (0.3)		
I’m not interested in any of them	2 (0.5)	8 (2.1)		

EFA and CFA samples were independent and non-overlapping. Values are presented as *n* (%). Chi-square tests were used to compare demographic distributions between the EFA and CFA samples. Regional background and economic status differed significantly between the two samples. The sexual interest variable included categories with small expected cell counts; therefore, the chi-square result for this variable should be interpreted cautiously. The original sexual interest item included a response option that combined sexual orientation and gender identity terminology and included outdated wording; therefore, this option was relabeled in the table as “Other/combined category as originally worded”.

### 2.6 Data analysis

The SPSS 21.0 (IBM Corp.; Armonk, NY, USA) and LISREL 8.80 (Scientific Software International, Inc.; Lincolnwood, IL, USA) package programs were used in the evaluation of the data after the data obtained from the research were coded by the researcher. The analytic strategy was informed by the original NMAS-BF development study, in which exploratory and confirmatory factor analyses were conducted in independent samples to evaluate the unidimensional structure of the brief form. Before conducting reliability and factor analyses, the two reverse-worded items of the NMAS-BF, corresponding to original NMAS items N2R and N7R, were reverse-coded before calculating the total score. In the Turkish item order used in this study, these corresponded to Items 3 and 5. Thus, higher scores consistently reflected higher levels of normative male alexithymia. The EFA and CFA datasets were screened for missing values, out-of-range responses, and response completeness. No missing responses were observed for the six NMAS-BF items in either dataset, and all item responses were within the expected 1–7 Likert-type response range. Chi-square tests were used to compare the demographic distributions of the independent EFA and CFA samples.

Internal consistency was evaluated using Cronbach’s alpha, corrected item-total correlations, and alpha-if-item-deleted values. Exploratory factor analysis was conducted using principal axis factoring to examine the latent structure of the Turkish NMAS-BF. Sampling adequacy was evaluated using the Kaiser–Meyer–Olkin coefficient and Bartlett’s test of sphericity. A one-factor solution was examined based on the theoretical structure of the original NMAS-BF, eigenvalues, scree plot inspection, explained variance, and factor interpretability. No rotation was applied because a single-factor solution was retained.

Confirmatory factor analysis was conducted in the independent CFA sample to test the one-factor structure identified in the EFA. Model fit was evaluated using multiple fit indices, including χ^2^/df, RMSEA, CFI, TLI/NNFI, SRMR, and GFI. α = 0.05 was considered statistically significant in all statistical analyses.

### 2.7 The ethical aspects of the study

Permission was obtained by e-mail from Ronald Levant, who had developed the NMAS-BF in 2019, prior to commencing the study. Ethics committee approval was obtained from the Karabük University Ethics Committee for Non-Invasive Clinical Studies (Date: October 7, 2019, Number: 77192459-050.99-E.10729) prior to application. Institutional permission was obtained from the rector’s office at the university where the study was conducted to conduct the study. Written information about the study was provided to the students who were to participate in the study, and their approval was obtained. (Informed volunteer consent form). After this, the demographic questionnaire and the NMAS-BF were presented, and the students were asked to provide responses to them in the manner most appropriate to them. The Helsinki Declaration was adopted during every phase of this study.

## 3 Results

### 3.1 Findings related to linguistic equivalence

The results of the Wilcoxon test and Spearman correlation findings obtained by applying the English and Turkish forms of the NMAS-BF to a group of 46 male students studying in the Department of English Language Teaching at a Faculty of Education in Eskişehir at 20-day intervals by the faculty members are shown in Table 1.

### 3.2 Findings related to reliability and validity analysis of the NMAS-BF

The demographic characteristics of the independent EFA and CFA samples are presented in Table 2. Chi-square comparisons indicated that the EFA and CFA samples were comparable in terms of birth year, number of siblings, family type, education level, and sexual interest. However, regional background and economic status differed significantly between the two samples. For the Turkish version of the six-item NMAS-BF, internal consistency was high, with a Cronbach’s α coefficient of 0.890. Corrected item-total correlations ranged from 0.617 to 0.770, and alpha-if-item-deleted values did not indicate that removing any item would improve the internal consistency of the scale; therefore, all six items were retained (Table 3).

**TABLE 3:** Item-level reliability indices, principal axis factor loadings, and CFA fit indices for the Turkish NMAS-BF.

Indicator	PAF loading	Communality	Corrected item-total correlation	α if item deleted
Item 1	0.784	0.614	0.737	0.867
Item 2	0.752	0.566	0.704	0.871
Item 3	0.832	0.692	0.770	0.861
Item 4	0.753	0.567	0.701	0.872
Item 5	0.784	0.615	0.729	0.867
Item 6	0.656	0.431	0.617	0.886
**EFA and reliability summary**			**Value**	
EFA sample size			376	
Extraction method			Principal axis factoring	
Rotation			None	
Number of retained factors			1	
Explained variance			58.09%	
KMO			0.850	
Bartlett’s test of sphericity			χ^2^(15) = 1299.214, *p* < 0.001	
Cronbach’s α			0.890	
CFA model fit				
CFA sample size			376	
χ^2^			20.66	
df			9	
*p*			0.014	
χ^2^/df			2.30	
RMSEA			0.059	
CFI			0.99	
TLI/NNFI			0.99	
SRMR			0.025	
GFI			0.98	
AGFI			0.96	

The two reverse-worded items of the NMAS-BF, corresponding to original NMAS items N2R and N7R, were reverse-coded before analysis; in the Turkish item order used in this study, these corresponded to Items 3 and 5. EFA was conducted using principal axis factoring. No rotation was applied because a single-factor solution was retained. CFA was conducted in an independent non-overlapping sample; therefore, CFA standardized loadings presented in Figure 2 are reported separately from the PAF loadings shown in this table. NNFI, Non-Normed Fit Index, equivalent to TLI; SRMR, standardized root mean square residual; AGFI, Adjusted Goodness of Fit Index.

**FIGURE 1:**
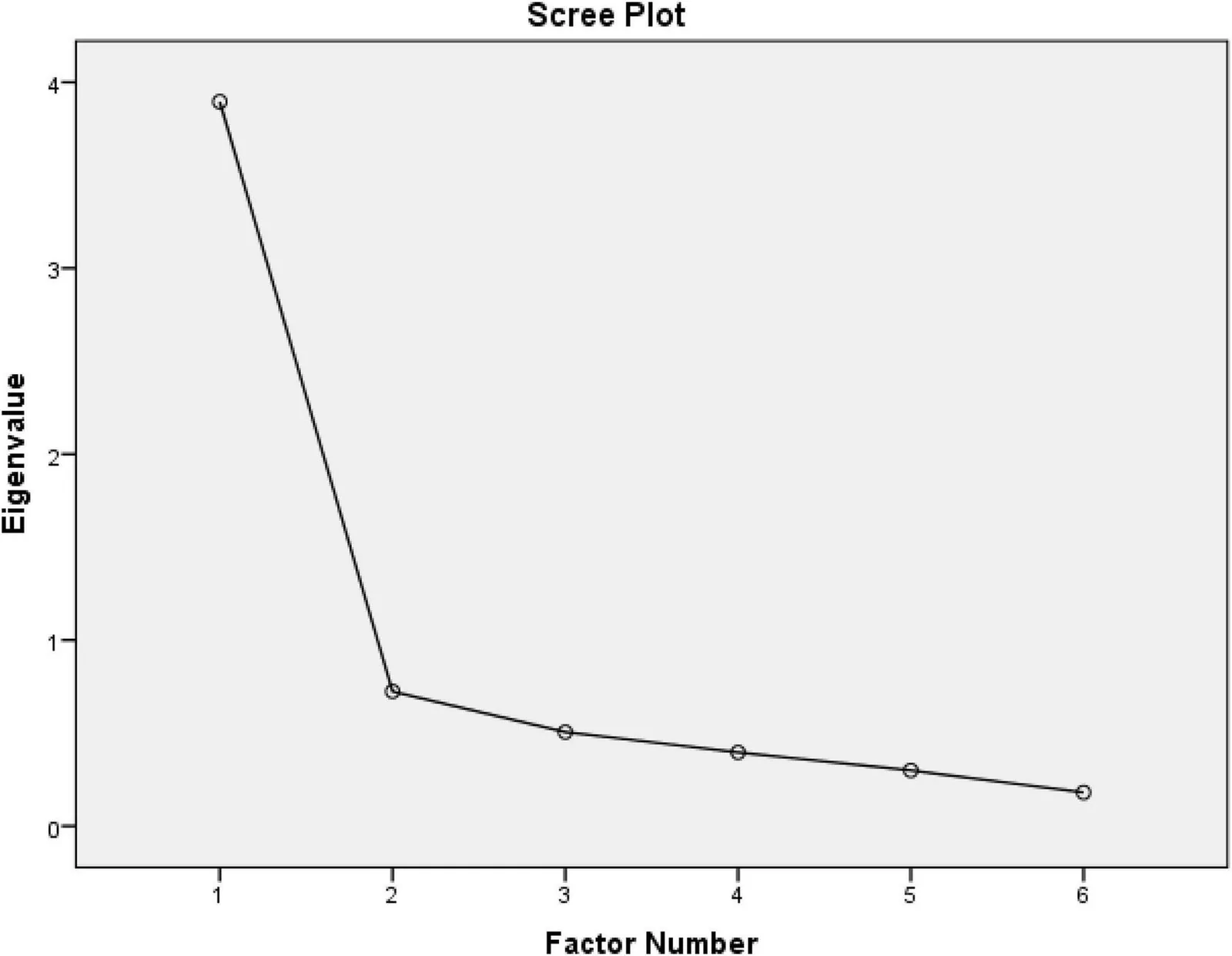
Scree plot supporting the single-factor structure of the Turkish NMAS-BF.

**FIGURE 2:**
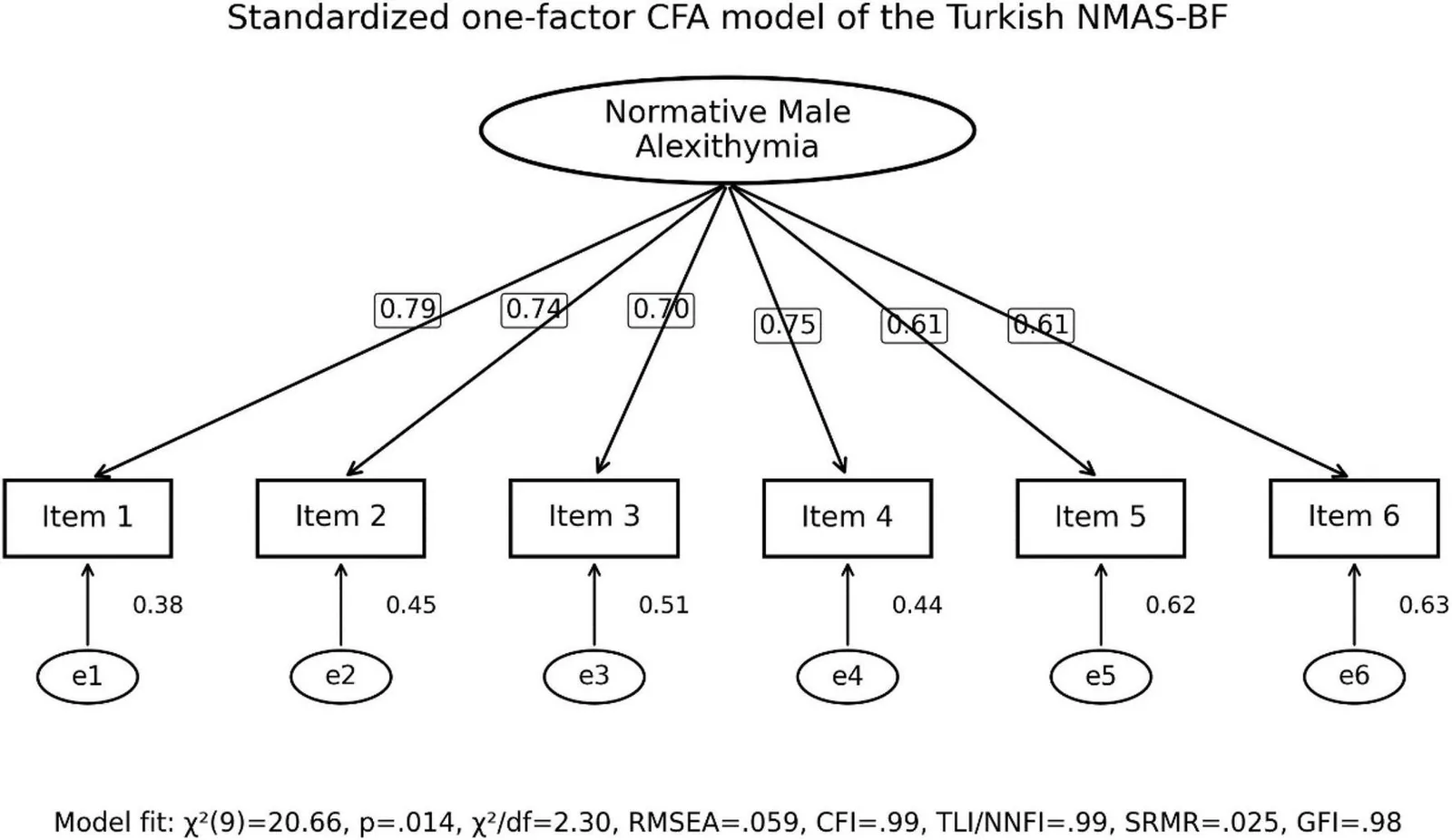
Standardized one-factor confirmatory factor analysis model of the Turkish NMAS-BF. Values on the paths represent standardized CFA factor loadings estimated in the independent CFA sample. These values are distinct from the principal axis factor loadings obtained in the EFA sample. e1–e6 represent item residuals. Model fit: χ^2^(9) = 20.66, *p* = 0.014, χ^2^/df = 2.30, RMSEA = 0.059, CFI = 0.99, TLI/NNFI = 0.99, SRMR = 0.025, and GFI = 0.98.

The data were suitable for factor analysis, as indicated by the Kaiser–Meyer–Olkin coefficient (KMO = 0.850) and Bartlett’s test of sphericity, χ^2^(15) = 1299.214, *p* < 0.001. Principal axis factoring supported a single-factor structure for the Turkish NMAS-BF. The factor loadings ranged from 0.656 to 0.832, and the single factor explained approximately 58.09% of the variance. No rotation was applied because a single-factor solution was retained (Figure 1, Table 3). Confirmatory factor analysis supported the one-factor model, χ^2^(9) = 20.66, *p* = 0.014, χ^2^/df = 2.30, RMSEA = 0.059, CFI = 0.99, TLI/NNFI = 0.99, SRMR = 0.025, and GFI = 0.98 (Figure 2, Table 3). The standardized factor loadings shown in Figure 2 are based on the CFA measurement model estimated in the independent CFA sample and therefore are not expected to be identical to the principal axis factor loadings obtained from the EFA sample.

## 4 Discussion

The present study adapted the NMAS-BF into Turkish and evaluated its psychometric properties among Turkish male university students. The main finding was that the Turkish NMAS-BF demonstrated satisfactory reliability and structural validity. Therefore, the present study should primarily be interpreted as a psychometric validation study of a brief measure designed to assess masculinity-related emotional restriction (Levant and Parent, 2019).

The reliability findings supported the internal consistency of the Turkish NMAS-BF, with a Cronbach’s α coefficient of 0.89. This finding is consistent with previous studies reporting strong reliability for the original NMAS and its brief form (Levant and Parent, 2019; Levant et al., 2006). The construct validity findings also supported the adequacy of the Turkish adaptation. This conclusion was based on principal axis factoring, item-level reliability indices, and confirmatory factor analysis in an independent validation sample. The KMO and Bartlett test results confirmed that the data were suitable for factor analysis, and the factor loadings indicated a strong item structure. Considering the six-item structure of the NMAS-BF, the EFA sample size was adequate for factor analysis, and this adequacy was further supported by the KMO value and Bartlett’s test of sphericity (Streiner, 1994). The single-factor structure explained approximately 58.09% of the variance using principal axis factoring, and the CFA results demonstrated acceptable to good model fit, with χ^2^/df = 2.30, RMSEA = 0.059, CFI = 0.99, TLI/NNFI = 0.99, SRMR = 0.025, and GFI = 0.98. Overall, these findings provide initial support for the internal consistency and structural validity of the Turkish NMAS-BF among male university students.

The single-factor structure of the Turkish NMAS-BF is theoretically meaningful when interpreted within the literature on normative male alexithymia and Turkish masculine socialization. Normative male alexithymia is theoretically linked to restrictive emotionality, which is shaped by masculine role socialization and expectations concerning strength, self-control, autonomy, and avoidance of vulnerability (Levant et al., 2006; Levant, 1992; Mahalik et al., 2003; O’Neil et al., 1986; O’Neil, 1981). In the Turkish context, masculine identity has been associated with expectations of strength, responsibility, social respectability, emotional toughness, self-control, authority, and avoidance of vulnerability (Boratav et al., 2014; Keskin, 2018; Tekkas Kerman and Betrus, 2019). Previous Turkish studies have also shown that masculinity, femininity, gender role conflict, and male role norms can be meaningfully examined in Turkish samples (Lease et al., 2009, 2013; Özkan and Lajunen, 2005). These expectations are conceptually consistent with the NMAS-BF items, which focus on difficulties in recognizing, naming, and expressing emotions (Levant and Parent, 2019). From this perspective, the Turkish NMAS-BF may capture a specific pattern of masculinity-related emotional restriction shaped and reinforced through gendered socialization processes.

These findings are conceptually relevant to masculinity research; however, the present study was designed as a psychometric validation study. Clinical outcomes, help-seeking behavior, mental health service utilization, and treatment engagement were not directly assessed. Accordingly, any implications for help-seeking or mental health service engagement should be considered theoretical and hypothesis-generating rather than empirically demonstrated by the present data. In addition, masculine socialization was discussed as a theoretical and cultural context rather than measured as a separate variable. Therefore, the findings should not be interpreted as evidence of causal or predictive relationships between Turkish masculine norms and normative male alexithymia, nor as evidence regarding clinical or service-use outcomes.

Several limitations should be considered when interpreting these findings. First, the study was conducted at a single university, which may limit the generalizability of the findings. Second, data were collected electronically; therefore, students without reliable internet access may have been excluded. Third, the use of self-report measures may have introduced social desirability bias, particularly given the gendered and culturally sensitive nature of emotional expression. Fourth, the sample consisted exclusively of male university students, and the findings may not generalize to older men, non-student populations, or men from different educational and socio-cultural backgrounds. Although the EFA and CFA samples were independent and comparable on several demographic variables, they differed significantly in regional background and economic status; therefore, these differences should be considered when interpreting the cross-sample structural findings. Fifth, psychiatric history, current psychiatric symptoms, depression, anxiety, trauma-related symptoms, substance use, and psychiatric treatment were not assessed; therefore, the potential influence of these variables on NMAS-BF scores could not be evaluated. Direct measures of masculine norm conformity, gender role conflict, family gender ideology, and social desirability were also not included, although these variables may have influenced participants’ responses. Sixth, the demographic item assessing sexual interest included terminology that combined sexual orientation and gender identity categories and included outdated wording. This item was used only descriptively, and future studies should assess sexual orientation using conceptually accurate and respectful response options. In addition, unlike the original NMAS-BF development study, the present adaptation did not examine convergent, concurrent, or incremental validity using measures such as the TAS-20, restrictive emotionality, conformity to masculine norms, or gender role conflict. Therefore, the nomological validity of the Turkish NMAS-BF should be evaluated in future studies. Finally, because the study did not assess help-seeking behavior, stigma, suicidal ideation, suicide attempts, clinical diagnosis, mental health service utilization, treatment engagement, or clinical outcomes, no conclusions can be drawn regarding these variables. Future studies should examine the Turkish NMAS-BF in broader age groups, non-student samples, community-based and clinical populations, and cross-cultural contexts. Further research should investigate whether normative male alexithymia is associated with emotional regulation, psychological well-being, and interpersonal functioning.

## 5 Conclusions

The Turkish version of the NMAS-BF demonstrated satisfactory internal consistency and structural validity among Turkish male university students, supporting its use as an initial measure of normative male alexithymia in this population. The findings suggest that the scale may be used in future research to assess masculinity-related emotional restriction within the Turkish cultural context. By situating normative male alexithymia within Turkish masculine socialization, this study provides a basis for future research on how culturally embedded masculine norms may relate to emotional awareness, emotional expression, and psychological functioning. However, the findings should not be interpreted as direct evidence regarding help-seeking behavior, stigma, suicidal ideation, suicide attempts, clinical diagnosis, mental health service utilization, treatment engagement, or clinical outcomes, as these variables were not assessed in the present study. Future research should examine the Turkish NMAS-BF in broader age groups, non-student samples, and clinical or community-based populations.

## Data Availability

The datasets presented in this article are not publicly available but may be obtained from the corresponding author upon reasonable request. Requests to access the datasets should be directed to İÖ, dr.iremozel@gmail.com.
